# *PTCH1* mutation promotes antitumor immunity and the response to immune checkpoint inhibitors in colorectal cancer patients

**DOI:** 10.1007/s00262-021-02966-9

**Published:** 2021-05-24

**Authors:** Yanni Wang, Huan Chen, Xi Jiao, Lihong Wu, Ying Yang, Jiao Zhang, Lijia Wu, Chang Liu, Na Zhuo, Shuang Li, Jifang Gong, Jian Li, Xiaotian Zhang, Xicheng Wang, Zhi Peng, Changsong Qi, Zhenghang Wang, Jie Li, Yan Li, Zhihao Lu, Henghui Zhang, Lin Shen

**Affiliations:** 1 Department of Gastrointestinal Oncology, Key Laboratory of Carcinogenesis and Translational Research, Ministry of Education/Beijing), Peking University Cancer Hospital and Institute, Fu-Cheng Road 52, Hai-Dian District, Beijing, 100142 China; 2 Genecast Biotechnology Co., Ltd, Wuxi, China; 3 Biomedical innovation center, Beijing Shijitan Hospital, and School of Oncology, Capital Medical University, Tieyi Road 10, Haidian District, Beijing, 100038 China

**Keywords:** *PTCH1*, Colorectal cancer, Immune checkpoint inhibitors, Biomarker

## Abstract

Immunotherapy has emerged as an effective therapeutic strategy for various cancers, including colorectal cancer (CRC), but only a subset of MSI-H patients can benefit from such therapy. Patched1 (*PTCH1*) is a frequently altered gene in CRCs and its mutations contribute to unregulated Hedgehog (Hh) signaling. In the study, we evaluated the association of *PTCH1* mutations with CRC immunity based on our single-center cohort and multiple cancer genomic datasets. Among 21 enrolled patients, six (28.6%) harbored a *PTCH1* mutation based on WES analyses. In CRC patients, the *PTCH1* mutation subgroup experienced a higher durable clinical benefit rate than the *PTCH1* wild-type subgroup (100% vs. 40%, *P* = 0.017). In addition, patients with the *PTCH1* mutation experienced greater progression-free survival (PFS, *P* = 0.037; HR, 0.208) and overall survival (OS, *P* = 0.045; HR, 0.185). A validation cohort from the MSKCC also confirmed the correlation between *PTCH1* mutation and better prognosis (*P* = 0.022; HR, 0.290). Mechanically, diverse antitumor immune signatures were more highly enriched in *PTCH1*-mutated tumors than in *PTCH1* wild-type tumors. Furthermore, *PTCH1*-mutated tumors had higher proportions of CD8 + T cells, activated NK cells, and M1 type macrophage infiltration, as well as elevated gene signatures of several steps in the cancer-immunity cycle. Notably, the *PTCH1* mutation was correlated with tumor mutational burden (TMB), loss of heterozygosity score, and copy number variation burden. Our results show that the mutation of *PTCH1* is a potential biomarker for predicting the response of CRC patients to immunotherapy.

**Supplementary Information:**

The online version contains supplementary material available at 10.1007/s00262-021-02966-9.

## Introduction

Colorectal cancer (CRC) is the third-most common cancer and the second leading cause of cancer-related death in the world [1]. Although early detection and systematic treatment have improved the survival rate of localized CRC, approximately 25% of patients still present with stage IV disease with a 5-year survival of only 14% [2]. Thus, the development of more effective treatments guided by actionable biomarkers for patients with this disease is urgently needed. In recent decades, the development of immune checkpoint inhibitors (ICIs) has revolutionized oncology, including CRC. In 2017, ICIs were approved for clinical use in patients with microsatellite instability-high (MSI-H) or deficient mismatch repair (dMMR) CRC. However, the MSI-H or dMMR subtype is present in approximately 5% of metastatic CRCs [3, 4], and only 30–40% of MSI-H/dMMR patients respond to ICIs [5–7], indicating an unmet clinical need for precision immunotherapy in CRC. Therefore, identifying additional biomarkers to precisely predict the response to ICIs in CRC patients is important.

The Hedgehog (Hh) signaling pathway, which regulates proliferation, angiogenesis, matrix remodeling, and stem cell renewal, plays an important role during tumorigenesis in CRC [8]. Upon secretion by cells, Hh ligands, such as sonic hedgehog (Shh), bind to Patched1 (*PTCH1*), thereby releasing the suppression of Smoothened (Smo), ultimately activating glioma-associated oncogene (Gli) transcription factors and promoting the transcription of Hh target genes. Recently, Hh signaling was reported to modulate the tumor microenvironment (TME) [9] by increasing immune checkpoint expression and promoting an inflammatory environment [10–12], thus indicating a potential association between Hh signaling and the response to ICIs. *PTCH1* is the most frequently altered Hh signaling regulator in CRC [13, 14], however the potential association of *PTCH1* with clinical outcomes for CRC patients receiving ICIs is unclear. Therefore, whether the mutation of *PTCH1* can predict the clinical outcome of ICI treatment was analyzed using data from our cohort and a publicly available cohort. Subsequently, we assessed immunogenic features based on *PTCH1* status to explore the possible underlying mechanism.

## Materials and methods

### Clinical cohorts

A total of 21 CRC patients treated with a PD-1/PD-L1 inhibitor combined with a CTLA-4 inhibitor between April 2011 and January 2017 at the Beijing Cancer Hospital were retrospectively analyzed. The tumor response was assessed by physicians using Response Evaluation Criteria in Solid Tumors (RECIST) version 1.1 criteria. Efficacy was defined as a durable clinical benefit (DCB, complete response [CR], partial response [PR], or stable disease [SD] lasting ≥ 6 months) or no durable benefit (NDB, progressive disease [PD] or SD lasting < 6 months) [15]. Another independent cohort consisting of 109 CRC patients treated with ICIs at Memorial Sloan Kettering Cancer Center (MSKCC) was also analyzed to validate the findings in our cohort. Clinical and genetic data were downloaded from the cBioPortal for Cancer Genomics at http://www.cbioportal.org/study?id=tmb_mskcc_2018. In addition, the Cancer Genome Atlas (TCGA) colorectal adenocarcinoma (COADREAD) database (www.cbioportal.org) including clinical and genomic data, as well as expression profile data, was further explored to determine the underlying mechanism.

### Genomic data analysis

DNA were extracted from formalin-fixed paraffin-embedded (FFPE) tissue specimens and matched white blood cell (WBC) samples of patients in our cohort. All specimens were collected from patients with written informed consent for their samples to be used in future translational researches including genomics, proteomics. Genetic alterations were subsequently analyzed according to standard principles. The tumor mutational burden (TMB) was measured in mutations per megabase (Mb) and was stratified into two groups, TMB-low (2–36 mutations/Mb) and TMB-high (> 37 mutations/Mb), as reported previously [16]. TMB level, loss of heterozygosity (LOH) score, and copy number variation (CNV) data were obtained from the TCGA database portal and analyzed according to a previously published study [17].

### MMR/MSI status detection

The MMR/MSI status was assessed according to the previous study [18]. In brief, the MMR status was identified using immunohistochemistry (IHC) for MMR protein such as monoclonal anti-mutL homolog 1, anti-mutS homolog 2, anti-mutS homolog 6, and PMS1 homolog 2. Deficient MMR tumors were defined as instability at two or more of these markers. Polymerase chain reaction (PCR) was performed to detect the MSI status, which assesses five microsatellite loci comprising BAT-25, BAT-26, D2S123, D5S346, and D17S250. MSI-H tumors were defined as instability at two or more of these markers.

### RNA expression profile analysis

Gene expression data were downloaded from the TCGA database portal and transformed into transcript per kilobase million (TPM) values. Subsequently, the log average of gene expression in TPM was used to quantify the enrichment level of three immune signatures, including GEP score (CXCR6, TIGIT, CD27, CD274, PDCD1LG2, LAG3, NKG7, PSMB10, CMKLR1, CD8A, IDO1, CCL5, CXCL9, HLA.DQA1, CD276, HLA-DRB1, STAT1,and HLA-E) [19, 20], immune cytolytic activity (GZMA and PRF1) [21], and IFN-*γ* signature (IFNG, CXCL10, CXCL9, IDO1, STAT1, and HLA-DRA) [22]. In addition, single-sample gene set enrichment analysis (ssGSEA) [23] and the CIBERSORT algorithm [24] were used to predict infiltration levels of multiple types of immune cells.

The immune response to tumors is triggered by critical steps referred to as the cancer-immunity cycle. In the present study, each step was quantified using immunogram scores (IGSs) [25] as follows: IGS1, T cell immunity; IGS2, tumor antigenicity; IGS3, T cell priming and activation; IGS4, T cell trafficking and infiltration; IGS5, antigen-presenting machinery; IGS6, inhibitory immune cell infiltration; IGS7, immune checkpoint molecules; and IGS8, inhibitory molecules. IGSs were assessed using Gene Set Variation Analysis (GSVA). The distributions of IGSs are presented as radar figures.

### Statistical analyses

All statistical analyses were performed using SPSS software version 23.0 for Windows and R 3.6.1. The survival function was estimated using Kaplan–Meier curves and the *P* value was determined using a log-rank test. Statistical heterogeneity was evaluated using the chi-squared test or Fisher’s exact test. Student’s *t* test was applied to determine the differences between two groups when data were normally distributed and continuous variables were compared using the Mann–Whitney U test. All reported *P* values were two-tailed and *P* values < 0.05 were considered statistically significant.

## Results

### Patient characteristics

A total of 21 patients from our center were included in the analysis. Baseline characteristics are summarized in Table 1. The median age was 44 years (range, 14–75 years) and 14 (66.7%) patients were male. Among the 21 patients, five experienced CR or PR, eight patients achieved SD, and eight patients had PD, resulting in a DCB rate of 57.1% and NDB rate of 42.9%. The tumors of 18 patients (85.7%) were tested for MSI status using PCR or MMR status using IHC; 16 (88.9%) were MSI-H or dMMR. Of these 16 patients with MSI-H/dMMR tumors, six (37.5%) had *PTCH1* mutation. However, *PTCH1* mutation was not detected in MSS/pMMR tumors. The median TMB value was 38.1 mutations/Mb (range, 2.3–220.3 mutations/Mb).

**Table 1:** Patient’s characteristics

Characteristics value^a^	Number	PTCH1-MUT	PTCH1-WT	*P*
	(*n* = 21)	(*n* = 6)	(*n* = 15)	
Age at diagnosis(year)				
Median age (range)	44 (14–75)	28.5 (15–31)	48 (33–75)	
Gender, *N* (%)				0.061
Male	14 (66.67%)	6 (100.00%)	8 (53.33%)	
Female	7 (33.33%)	0	7 (46.67%)	
Histopathology, *N* (%)				1.000
Adenocarcinoma	19 (90.48%)	6 (100.00%)	13 (86.67%)	
Others	2 (9.52%)	0	2 (13.33%)	
Treatment option, *N* (%)				1.000
Anti-PD-(L)1	19 (90.48%)	6 (100.00%)	13 (86.67%)	
Anti-PD-(L)1 + anti-CTLA-4	2 (9.52%)	0	2 (13.33%)	
Best response, *N* (%)				0.140
Complete response	2 (9.52%)	1 (16.67%)	1 (16.67%)	
Partial response	3 (14.28%)	1 (16.67%)	2 (13.33%)	
Stable disease	8 (38.10%)	4 (66.66%)	4 (26.67%)	
Progressive disease	8 (38.10%)	0	8 (53.33%)	
Response group, N (%)				0.017
Durable clinical benefit (DCB)	12 (57.14%)	6 (100.00%)	6 (40.00%)	
No durable benefit (NDB)	9 (42.86%)	0	9 (60.00%)	
Tumor MSI/MMR status, N (%)				0.269
MSI-H/dMMR	16 (76.19%)	6 (100.00%)	10 (66.67%)	
pMMR	2 (9.52%)	0	2 (13.33%)	
Not available	3 (14.29%)	0	3 (20.00%)	

^a^*P* values derived from Fisher’s exact tests between the two groups

### Predictive role of *PTCH1* and TMB in the study cohort

**Fig. 1:**
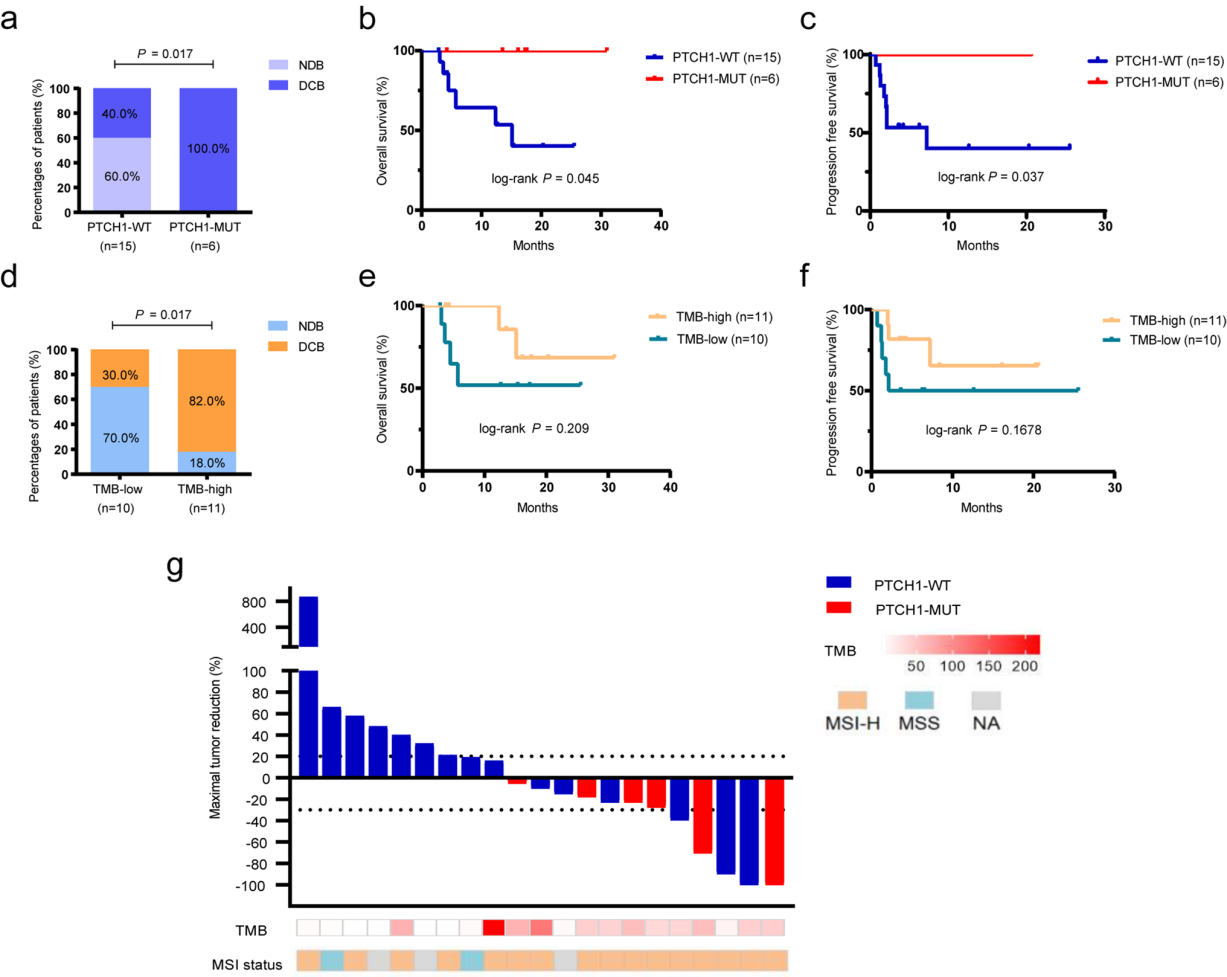
PTCH1 and TMB status correlate with the response to ICI treatment in our cohort** a** Comparison of the DCB rates in the *PTCH1* mutation and *PTCH1* wild-type groups. **b** and **c** Kaplan–Meier curves comparing OS (**b**) and PFS (**c**) in patients with the *PTCH1* mutation and wild-type *PTCH1* in our cohort. **d** Comparison of the DCB rates in the TMB-low and TMB-high groups. **e** and **f** Kaplan–Meier curves comparing OS (**e**) and PFS (**f**) of the TMB-high and TMB-low patients. The cutoff value for TMB-high and TMB-low was defined as 37 mutations/Mb. **g** Waterfall plot representing the best change from baseline in sum of longest target lesion diameters per patient based on *PTCH1*, TMB, and MSI status. *PTCH1*, Patched1 gene; DCB, durable clinical benefit; OS, overall survival; PFS, progression-free survival; TMB, tumor mutational burden; MSI, microsatellite instability

First, whether the *PTCH1* mutation was associated with the clinical benefit from ICIs in CRC patients was analyzed. The DCB rate was higher in patients with the *PTCH1* mutation than in *PTCH1* wild-type patients (100% vs. 40%, *P* = 0.017; Fig. 1a), this is mainly due to higher proportion of SD lasting ≥ 6 months in *PTCH1* mutation group. In addition, a favorable prognosis (better OS/PFS) was identified in the population with the *PTCH1* mutation (OS, *P* = 0.045, HR, 0.185, 95% CI, 0.035–0.965; PFS, *P* = 0.037 HR, 0.208, 95% CI, 0.048–0.911; Fig. 1b, c). Among 16 MSI-H/dMMR patients, the *PTCH1* mutation was also associated with improved OS (*P* = 0.0607, HR, 0.149, 95% CI, 0.020–1.090) and PFS (*P* = 0.057, HR, 0.178, 95% CI, 0.030–1.055) compared with *PTCH1* wild-type patients, although with marginal significance (Supplementary Fig. S1a and b). Next, the predictive value of TMB was evaluated in our cohort because it is considered a predictive biomarker in multiple cancers [26]. Unfortunately, a statistically significant difference was not observed in the DCB rates or survival outcomes between TMB-high and TMB-low patients when using the cutoff value of 37 as previously suggested (Fig. 1d–f). Waterfall plots depicting the tumor change from baseline in individual patients based on their *PTCH1* or TMB status are shown in Fig. 1g.

### Validation of *PTCH1* and TMB as biomarkers in the MSKCC CRC ICI cohort

**Fig. 2:**
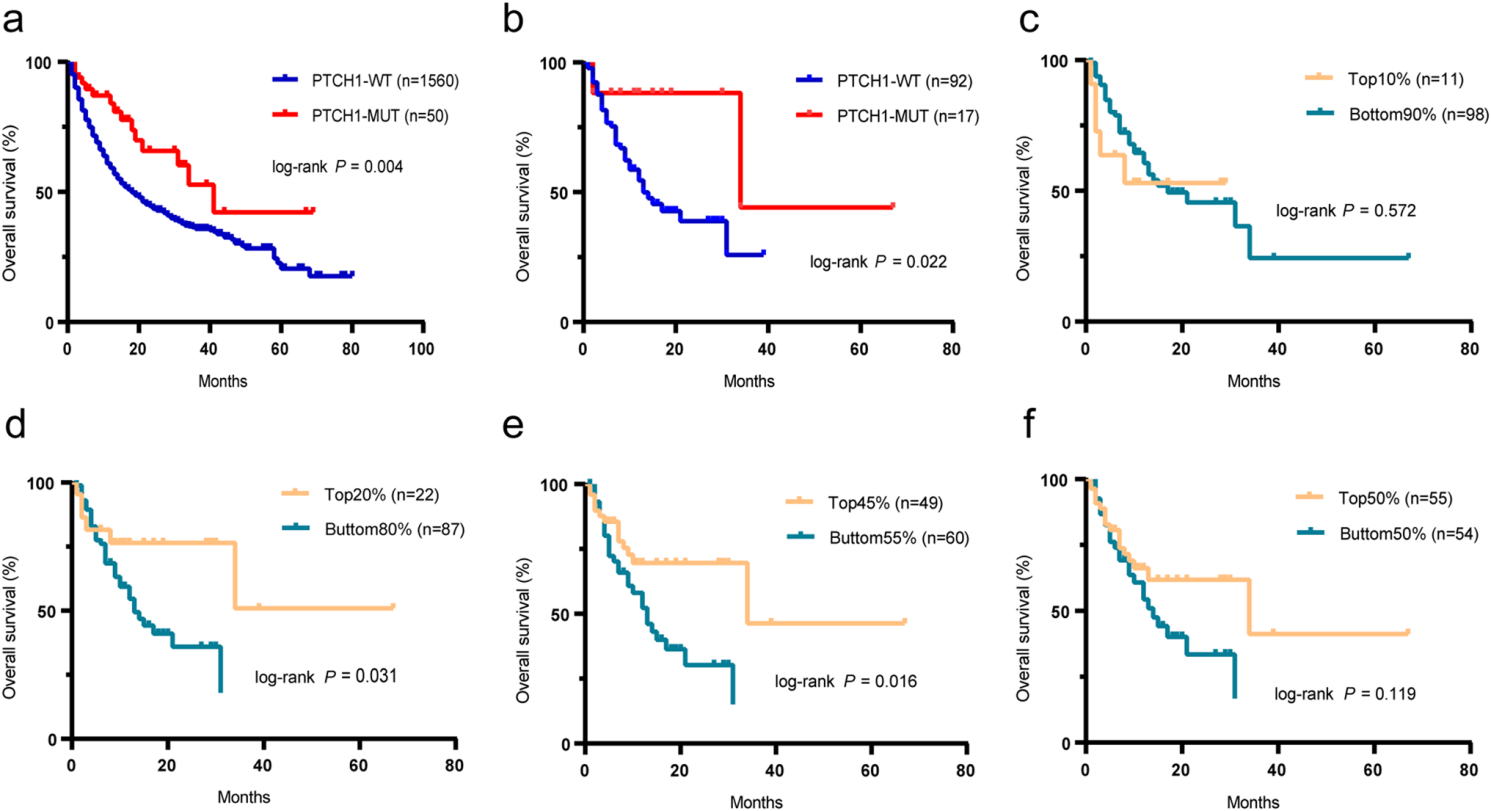
Survival analysis of patients stratified based on *PTCH1* status or TMB in the MSKCC cohort** a** and **b** OS in patients with and without the *PTCH1* mutation in the MSKCC pan-cancer (**a**) and MSKCC CRC cohort (**b**). **c**-**f** OS curves were plotted for the patients stratified based on the TMB level in the MSKCC cohort. The cutoffs used in this cohort were the top 10% (c), 20% (d), 45% (e), and 50% (f).

To validate the above results, another cohort (MSKCC ICI) consisting of 1,610 patients treated with ICIs, including 109 CRC patients, was used. In both the pan-cancer cohort and a subset of CRC patients, those harboring the *PTCH1* mutation also had better survival outcomes (*P* = 0.004, HR, 0.485, 95% CI, 0.337–0.697; *P* = 0.022, HR, 0.29, 95% CI, 0.139–0.607; Fig. 2a, b). Consistent with a previous study [26], a higher TMB (highest 25%) was associated with better OS. However, statistical significance was not always reached when using cutoff values outside of appropriate ranges (Fig. 2c–f, cutoff values of 10%, 20%, 45%, and 50%). Together with the findings from our cohort, these results indicate the robustness of TMB as a predictive biomarker for predicting benefit from ICIs.

### Correlation between the *PTCH1* mutation and immune-related signatures

**Fig. 3:**
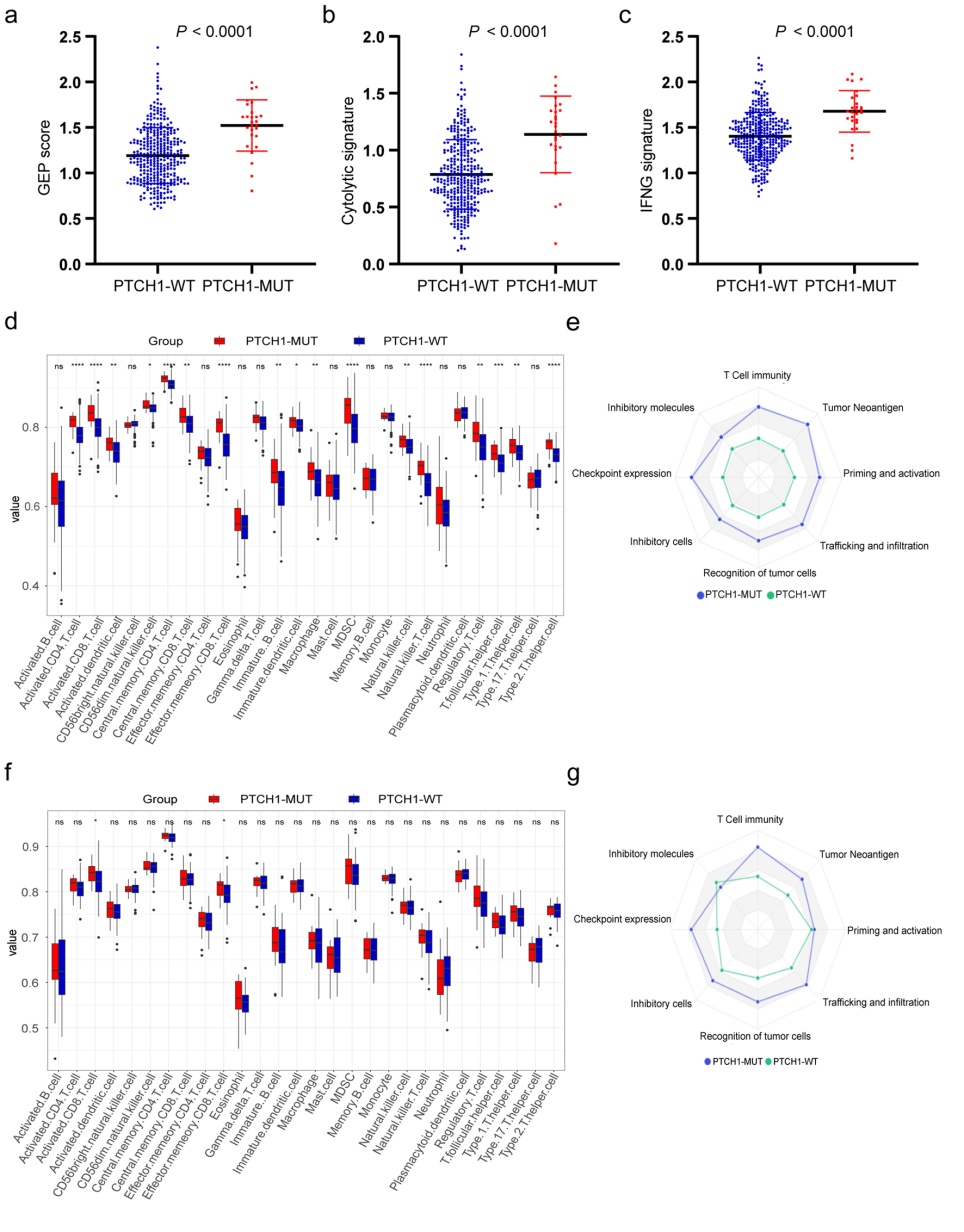
Correlation between *PTCH1* mutation and immune-related signatures in the TCGA cohort a–c Quantitative analysis of GEP scores (**a**), Cytolytic (**b**) and IFNG (**c**) signatures in the *PTCH1* mutation and *PTCH1* wild-type patients based on the TCGA COADREAD database. **d** and **f** Boxplots show the abundance of ssGSEA-derived immune cells in multiple cell subsets based on *PTCH1* mutation status in the TCGA COADREAD cohort (**d**) and its MSI-H subgroup (**f**). **e** and **g** Eight axes of an immunogram involving the cancer-immunity cycle were plotted based on the *PTCH1* mutation status in the TCGA COADREAD cohort (**e**) and its MSI-H subgroup (**g**). The value of the immunogram score (IGS) was calculated using GSVA. GEP, gene expression signature; GSVA, Gene Set Variation Analysis

Based on the above observations, we hypothesized that *PTCH1* status influenced the shaping of the TME. To verify this hypothesis, the T cell-inflamed gene expression signature (GEP) consisting of 18 genes indicative of a T cell activated tumor, which was shown to predict the response to ICIs [27], was analyzed. Based on the expression profile from the TCGA cohort, *PTCH1*-mutated tumors displayed a higher GEP signature (Fig. 3a). Furthermore, greater enrichment of IFN-*γ* and cytolytic signatures in the *PTCH1* mutation subgroup were observed (Fig. 3b, c).

To characterize the immune infiltration landscape of the *PTCH1* mutation and wild-type subgroups, ssGSEA scores from the 28 immune-related signatures were used to quantify the relative abundance of 28 immune cell types. *PTCH1*-mutated tumors had higher proportions of CD8^+^ T cells and activated NK cells, indicating the induction of an antitumor response (Fig. 3d). Similar results were obtained when using the CIBERSORT algorithm (Supplementary Fig. S2). Specifically, in the MSI-H population, CD8^+^ T cells and effector memory CD8^+^ T cells were enriched in *PTCH1*-mutated tumors (Fig. 3f). To obtain a more comprehensive understanding of the relationship between *PTCH1* mutation and tumor-immunity interactions, an immunogram was used to visualize the general cancer immunity status in the *PTCH1* mutation and wild-type patients. The steps in the cancer-immunity cycle were enhanced in *PTCH1*-mutated tumors in the entire TCGA COADREAD cohort and its MSI-H subgroup (Fig. 3e, g), indicating that the mutation of *PTCH1* in CRC potentially facilitates immune cell infiltration, partially accounting for the better response to ICIs in the *PTCH1* mutation subgroup.

### Correlations between *PTCH1* mutation and genomic parameters

**Fig. 4:**
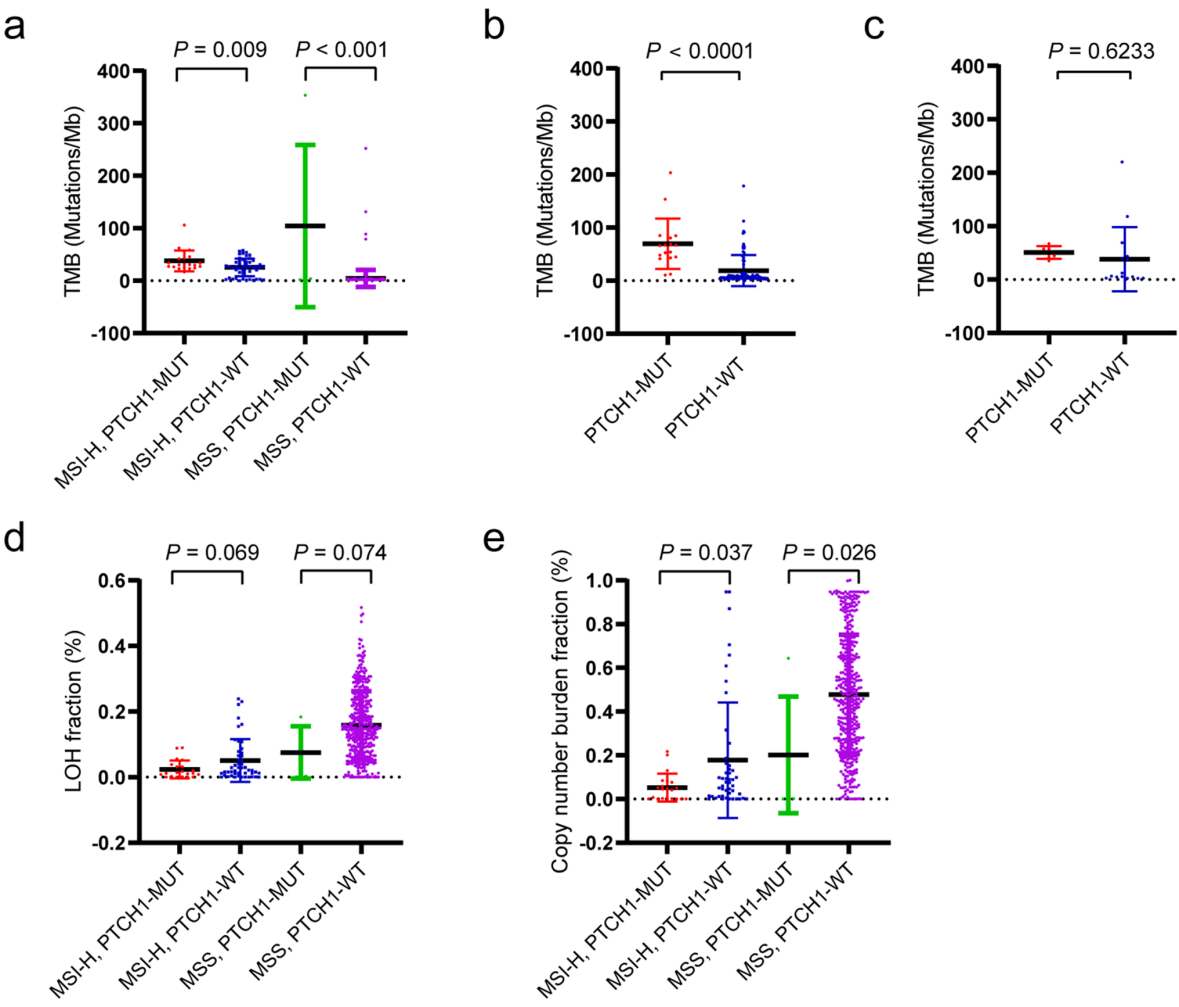
Correlations between *PTCH1* mutation and genomic parameters **a** Comparison between the *PTCH1* mutation and wild-type subgroups in MSI-H and MSS populations based on the TCGA COADREAD database. **b** Analysis of TMB level in the *PTCH1*-mutated and wild-type tumors in the MSKCC cohort. **c** Analysis of TMB level in the *PTCH1*-mutated and wild-type tumors in our cohort. **d** and **e** Comparison of LOH (**d**) and CNV burden (**e**) between the *PTCH1* mutation and wild-type subgroups in the MSI-H and MSS populations based on the TCGA COADREAD database. LOH, loss of heterozygosity; CNV, copy number variation

In a previous study, *PTCH1*-driven skin basal cell carcinoma showed a high level of TMB [28], thus, the TMB levels in the *PTCH1* mutation and wild-type groups from our cohort and database were evaluated. The analysis confirmed that the *PTCH1* mutation subgroup had a higher TMB than the *PTCH1* wild-type subgroup in both MSI-H and MSS populations in the TCGA database (Fig. 4a) and MSKCC cohort (Fig. 4b). However, patients with the *PTCH1* mutation in our cohort did not show a higher TMB level; this may be explained by the relatively small sample size (Fig. 4c). Notably, a lower LOH and CNV burden were identified in patients with the *PTCH1* mutation regardless of MSI status (Fig. 4d, e). The above results imply that the mutation of *PTCH1* may be indicative of genomic tumor features.

## Discussion

Our study based on this small cohort of patients shows the *PTCH1* mutation was associated with better survival outcomes (OS/PFS) and DCB rates among patients who received ICIs, which is partially attributable to a higher proportion of SD lasting ≥ 6 months in *PTCH1* mutation group. Potentially, the *PTCH1* mutation established a favorable immune contexture of CRC, thus sensitizing tumors to immunotherapy.

The main challenge of ICI treatment in CRC patients is that only 30–40% of MSI-H/dMMR CRCs respond to ICIs [5–7], and the underlying molecular mechanisms of this clinical observation are not well known. Although quantifying TMB and MSI density may precisely stratify MSI-H patients for ICI treatment [16, 29], the present technical and practical barriers limit the use of these strategies in clinical practice. Furthermore, mixed results of the predictive role of TMB based on different cutoff values were elicited in our and MSKCC cohorts, indicating that clarifying the TMB cutoff values specific to CRC and its MSI-H subtype to identify who will receive optimal benefit is important. Other than TMB, two independent cohorts showed prolonged OS in patients with the *PTCH1* mutation. The clinical benefit analysis also indicated that patients with a *PTCH1* mutation experienced a higher DCB rate than *PTCH1* wild-type patients. The results of the present study suggest that *PTCH1* status could also function as a predictive biomarker for response to ICI treatment, in addition to TMB and MSI density. Moreover, The detection of *PTCH1* mutations may provide a more accessible way to stratify MSI-H/dMMR CRC patients for immunotherapy, though a study using a larger cohort would be necessarty to validate these findings.

PTCH1 plays a pivotal role in regulating Hh signaling, inactivating mutations that can activate the expression of Hh target genes in an unregulated manner [8]. The involvement of Hh signaling has been associated with tumor development, recurrence, metastasis, and TME regulation in CRC. In recent studies, Hh-driven skin basal cell carcinoma had a significantly high TMB level with an average of 65 mutations per Mb [28, 30], indicating that Hh-driven tumors likely represent an immunogenic entity. Similarly, tumors harboring the *PTCH1* mutation had a higher level of TMB in the present study. Recently, Lin A et al. [14] has found that *PTCH1* deficiency leads to the increased secretion of cytokines that promote tumor-antigen presentation, facilitate T lymphocytes infiltration. These notions, further support our findings that tumors with *PTCH1* mutation display a “hot” TME phenotype, with a high immune infiltration and TMB/neoantigen level, partially explaining why patients with a *PTCH1* mutation respond well to ICIs.

The present study has several limitations. First, this was a retrospective study that included patients treated with both single-agent PD-1/PD-L1 blockade and combinational immunotherapy. Second, because MSI detection was not the standard-of-care at the time when some of these patients were treated, three patients lacked MSI status information and two patients were identified with dMMR. Third, experimental information to determine the impact of *PTCH1* on immune contexture was lacking. Future investigations based on *in vitro* and *in vivo* experiments and larger clinical cohorts are warranted to confirm these findings. However, the present study based on multiple cohorts was the first to demonstrate the value of *PTCH1* for predicting the response to ICIs.

In the present study, ICI treatment was shown to be more effective in CRC patients with a *PTCH1* mutation. Although we did not have adequate samples in our cohort, it is an important finding that the *PTCH1* mutation is characterized by activation of the immune microenvironment and may be used to predict the response of CRC patients to ICIs. Further verification is needed in additional large cohorts.

## Supplementary Information

Below is the link to the electronic supplementary material.

Supplementary file1 (PDF 298 KB)

Supplementary file2 (PDF 373 KB)

## Data Availability

All data generated or analyzed during this study are included in this published article and its supplementary information files.

## Abbreviations

CNV: Copy number variation
CR: Complete response
CRC: Colorectal cancer
DCB: Durable clinical benefit
dMMR: Deficient mismatch repair
FFPE: Formalin-fixed paraffin-embedded
GEP: Gene expression signature
Gli: Glioma-associated oncogene
Hh: Hedgehog
ICIs: Immune checkpoint inhibitors
LOH: Loss of heterozygosity
MSI-H: Microsatellite instability-high
OS: Overall survival
PD: Progressive disease
PFS: Progression-free survival
PR: Partial response
PTCH1: Patched1
SD: Stable disease
Shh: Sonic hedgehog
ssGSEA: Single-sample gene set enrichment analysis
TMB: Tumor mutational burden
TME: Tumor microenvironment
TPM: Transcript per kilobase million
WBC: White blood cell
