# Supplementary material for: PTCH1 mutation promotes antitumor immunity and the response to immune checkpoint inhibitors in colorectal cancer patients
Source: Cancer Immunol Immunother. 2021 May 24;71(1):111–20. doi: 10.1007/s00262-021-02966-9 (PMC8738454; doi:10.1007/s00262-021-02966-9)
Supplement: Supplementary file 1 — Supplementary file1 (PDF 298 KB) [file 262_2021_2966_MOESM1_ESM.pdf]

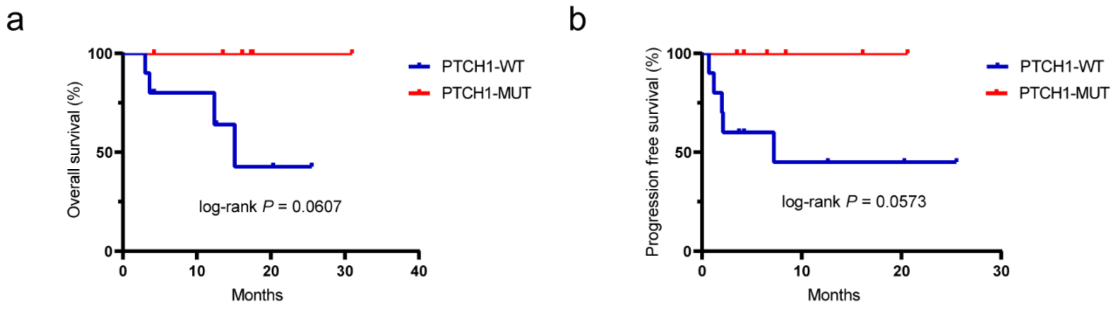

Fig. S1 Kaplan-Meier survival curves of OS (a) and PFS (b) comparing *PTCH1* wild-type and *PTCH1* mutation patients in the MSI-H populations of our cohort.
