# Supplementary material for: PTCH1 mutation promotes antitumor immunity and the response to immune checkpoint inhibitors in colorectal cancer patients
Source: Cancer Immunol Immunother. 2021 May 24;71(1):111–20. doi: 10.1007/s00262-021-02966-9 (PMC8738454; doi:10.1007/s00262-021-02966-9)
Supplement: Supplementary file 2 — Supplementary file2 (PDF 373 KB) [file 262_2021_2966_MOESM2_ESM.pdf]

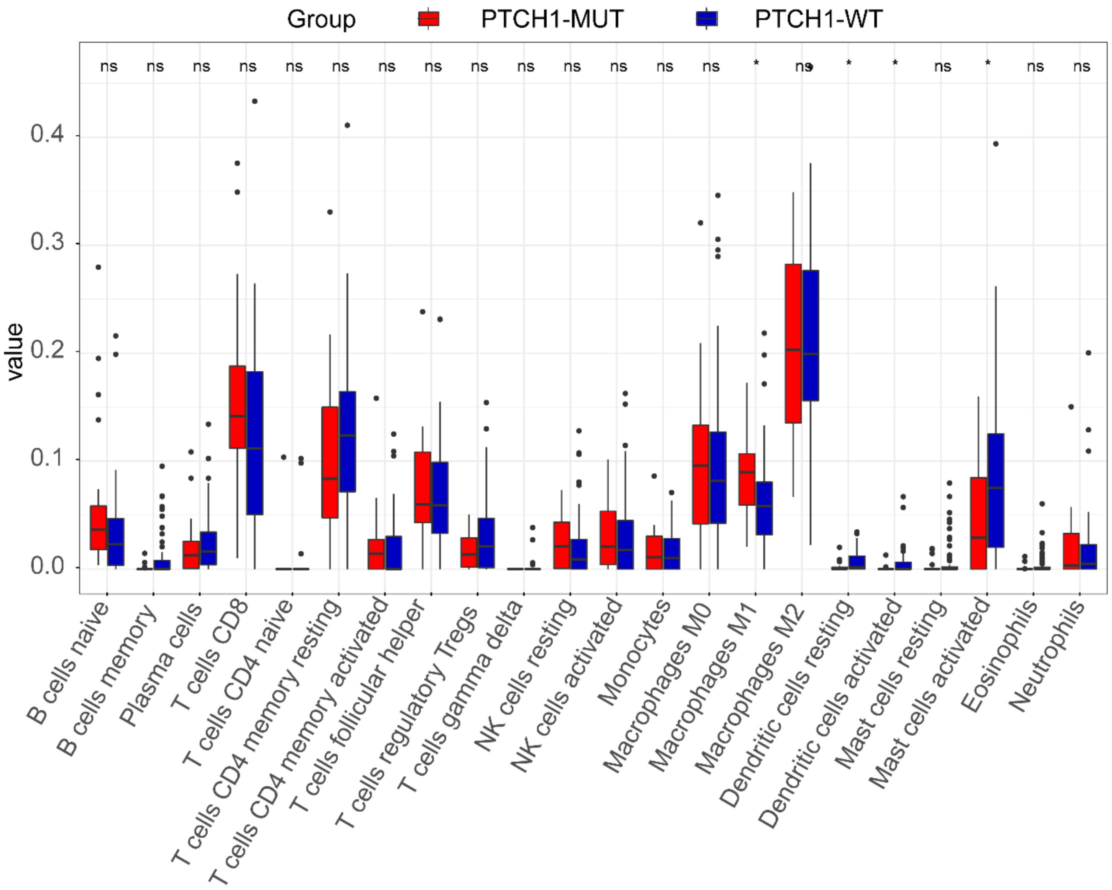

Fig.S2 The boxplot shows the abundance of CIBERSORT-derived immune cells in multiple cell subsets based on *PTCH1* status in the TCGA cohort.
